# *Antrodia cinnamomea* induces autophagic cell death via the CHOP/TRB3/Akt/mTOR pathway in colorectal cancer cells

**DOI:** 10.1038/s41598-018-35780-y

**Published:** 2018-11-27

**Authors:** Dai-Hua Tsai, Cheng-Han Chung, Kung-Ta Lee

**Affiliations:** 1 Department of Biochemical Science and Technology, National Taiwan University, Taipei, 10617 Taiwan; 2 Yong Teng biotechnology Co., Ltd., New Taipei City, 22180 Taiwan

## Abstract

*Antrodia cinnamomea*, a well-known traditional medicine used in Taiwan, is a potent anticancer drug for colorectal cancer, but the upstream molecular mechanism of its anticancer effects remains unclear. In this study, *A. cinnamomea* extracts showed cytotoxicity in HCT116, HT29, SW480, Caco-2 and, Colo205 colorectal cancer cells. Whole-genome expression profiling of *A. cinnamomea* extracts in HCT116 cells was performed. *A. cinnamomea* extracts upregulated the expression of the endoplasmic reticulum stress marker CHOP and its downstream gene TRB3. Moreover, dephosphorylation of Akt and mTOR as well as autophagic cell death were observed. Gene expression and autophagic cell death were reversed by the knockdown of CHOP and TRB3. Autophagy inhibition but not apoptosis inhibition reversed *A. cinnamomea*-induced cell death. Finally, we demonstrated that *A. cinnamomea* extracts significantly suppressed HCT116 tumour growth in nude mice. Our findings suggest that autophagic cell death via the CHOP/TRB3/Akt/mTOR pathway may represent a new mechanism of anti-colorectal cancer action by *A. cinnamomea*. *A. cinnamomea* is a new CHOP activator and potential drug that can be used in colorectal cancer treatment.

## Introduction

Colorectal cancer is one of the most common cancers (an estimated 1.36 million new cases occurred in 2012) worldwide^1^, and statistics show that the incidence rates of colorectal cancer are increasing in many countries, such as Latin America, Asia, and Eastern Europe^2^. Although there are many treatment strategies for colorectal cancer, such as chemotherapy, surgery, radiation therapy, targeted therapy, and immunotherapy, nearly 0.7 million people are estimated to have died from colorectal cancer in 2012 worldwide^1^. For this reason, finding new drugs against colorectal cancer is urgent.

Over the past decades, natural-source cancer drugs have sufficiently served to combat cancer, and over 60% of the anticancer agents approved since 1940 that are available for use can be traced to a natural product^3^. Paclitaxel is one of the most well-known natural products in cancer treatment. In addition, previous studies indicated that many natural products, such as curcumin, epigallocatechin gallate, and shikonin, are potent drug candidates for cancer treatment^3–5^.

*Antrodia cinnamomea* (also known as *Antrodia camphorata*), famed as “the ruby of the forest” in Taiwan, is a rare mushroom that grows only on the native Taiwanese tree *Cinnamomum kanehirai*^6^. *A. cinnamomea* has been used in traditional medicine for hundreds of years to treat discomforts caused by alcohol consumption, exhaustion, diarrhoea, abdominal pain, hypertension and cancer^6–8^. Several researchers have reported on the different biological activities of *A. cinnamomea*, which include anticancer activities against liver cancer^9^, colorectal cancer^10^, lung cancer^11^, breast cancer^12^, and leukaemia^13^; anti-inflammatory and immunomodulatory effects^14^; hepatoprotective activities^15^; and antioxidant activities^16^.

In colorectal cancer, *A. cinnamomea* showed potent anticancer activities *in vitro*^10,17,18^ and *in vivo*^19,20^. Treatment with *A. cinnamomea* extracts alone or combination with amphotericin B induced cell cycle arrest in HT29 human colorectal cancer cells^17,20^. Treatment with SY-1, a compound purified from *A. cinnamomea*, induced G0/G1 cell cycle arrest in Colo205 human colorectal cancer cells^10,19^. Treatment with *A. cinnamomea* also caused HT29 and Colo205 cells to undergo apoptotic cell death^10,17^. Furthermore, antroquinonol, a derivative of *A. cinnamomea*, suppresses colon cancer stem cell-like properties via targeting the PI3K/AKT/β-catenin signalling pathway^18^.

Although the expression of several genes has been shown to mediate the effects of *A. cinnamomea* in colorectal cancer, more evidence of the pharmacological mechanisms at the molecular level remains necessary for better understanding. Microarray technology and the associated bioinformatic tools have become widely used methods to investigate the molecular mechanisms of traditional Chinese medicines^21,22^. According to microarray gene expression profiles, Si-Wu-Tang, a traditional Chinese medicinal formula utilized for menstrual discomfort relief, was identified as a Nrf2 activator and suggested to be used as a nontoxic chemopreventive agent^23^. Gene expression profiles indicated that VI-28, a traditional Chinese medicinal formula originally designed to be an anti-aging health product, was shown to regulate innate and adaptive immune gene expression^24^. Microarray analysis results showed that a new immunomodulatory protein, ACA, purified from *A. cinnamomea* exhibited TLR2-dependent NF-KB activation in murine macrophages^25^. We presume that whole-genome expression profiling can provide deep insights into the molecular mechanisms mediating the anticancer activity of *A. cinnamomea* in colorectal cancer.

The aims of this work were to examine whether *A. cinnamomea* can help fight against colorectal cancer and identify the molecular mechanisms underlying its anticancer activity. First, we evaluated the antitumour activity of *A. cinnamomea* in five colorectal cancer cell lines. Then, next-generation sequencing (NGS) was used to analyse gene expression changes after *A. cinnamomea* treatment. Finally, we examined the expression of genes identified using whole-genome expression profiling and confirmed the molecular mechanisms underlying the anticancer effects of *A. cinnamomea* in colorectal cancer.

## Results

### *A. cinnamomea* extract isolation

**Figure 1:**
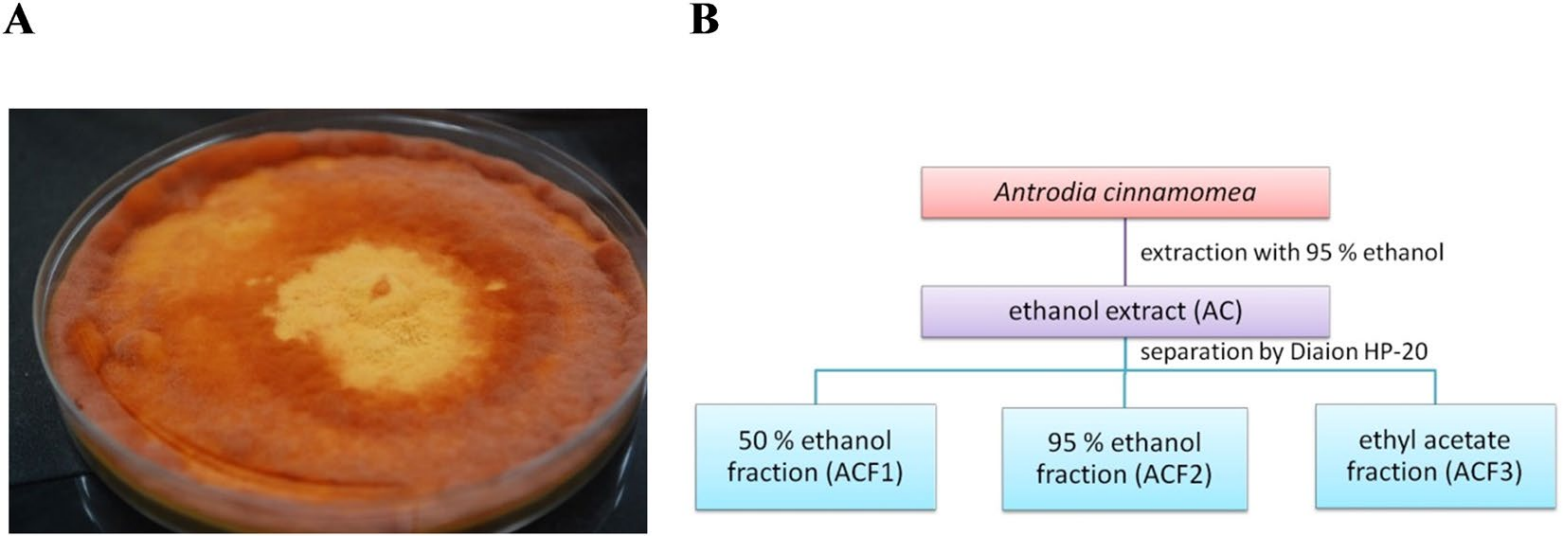
*A. cinnamomea* extract isolation. (**A**) Morphological observations of the *A. cinnamomea* fruiting bodies analysed in this study. (**B**) Scheme depicting the methodology used to obtain AC, ACF1, ACF2, and ACF3.

The *A. cinnamomea* fruiting bodies used in this study are shown in Fig. 1A. After extraction by ethanol and separation by Diaion HP-20, the *A. cinnamomea* extracts AC, ACF1, ACF2, and ACF3 were obtained (Fig. 1B).

### *A. cinnamomea* extracts inhibit colorectal cancer cell viability

**Figure 2:**
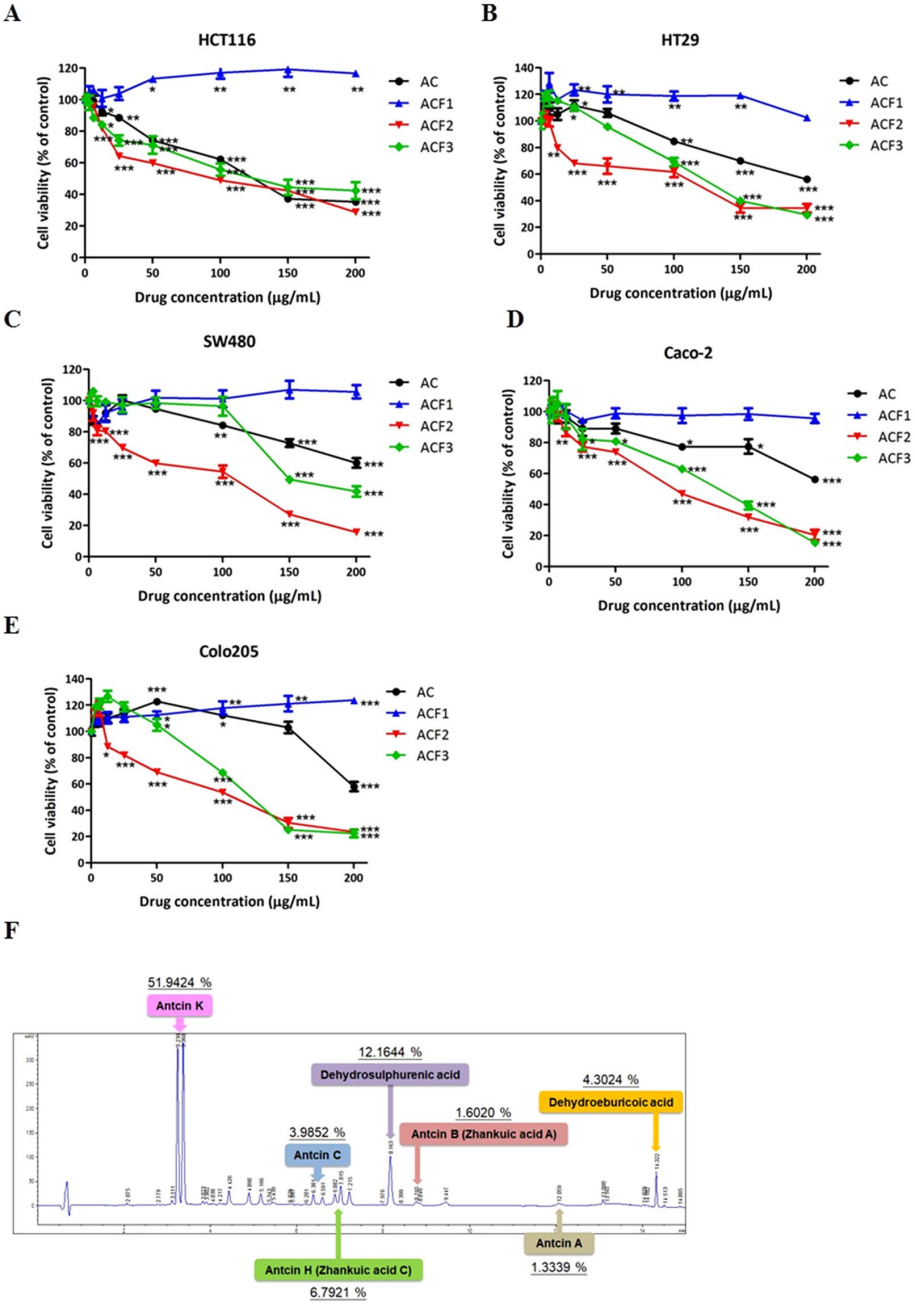
Effect of *A. cinnamomea* extract treatment on the cell viability of colorectal cancer cell lines and HPLC chemical fingerprinting. (**A**) HCT116, (**B**) HT29, (**C**) SW480, (**D**) Caco-2 and (**E**) Colo205 cells were treated with AC, ACF1, ACF2, and ACF3 for 48 h. The cell viability was analysed by the MTS assay and expressed as cell viability (% control). All results are expressed as the mean ± standard deviation of three independent experiments. P values of statistical significance are represented as *p < 0.05, **p < 0.005 and ***p < 0.0005. (**F**) HPLC chemical fingerprint of ACF2. The compounds are antcin K, antcin C, antcin H (Zhankuic acid C), dehydrosulphurenic acid, antcin B (Zhankuic acid A), antcin A, and dehydroeburicoic acid.

To investigate whether *A. cinnamomea* has an anticancer effect on colorectal cancer, the 3-(4,5-dimethylthiazol-2-yl)-5-(3-carboxymethoxyphenyl)-2-(4-sulphophenyl)-2H-tetrazolium (MTS) assay was performed to evaluate its cytotoxic role on HCT116, HT29, SW480, Caco-2 and Colo205 human colorectal cancer cells. As shown in Fig. 2, after 48 h of treatment, AC, ACF2, and ACF3 inhibited cell viability in a dose-dependent manner in all five cell lines. However, ACF1 had no cytotoxic effect. ACF2 showed the strongest cytotoxicity in HCT116, HT29, SW480, Caco-2 and Colo205 cells with IC_50_ values of 33.21 ± 13.25, 49.28 ± 34.23, 98.53 ± 10.63, 84.89 ± 7.94, and 55.28 ± 10.53 μg/ml, respectively. Direct trypan blue exclusion cell counts were performed to confirm the results of the MTS assay. The cell count results for HCT116 cells treated with *A. cinnamomea* extracts were consistent with the IC_50_ values determined by the MTS assay (Supplementary Figure 1). As shown in Fig. 2F, the HPLC fingerprint of ACF2 identified seven major components and their respective structures: antcin K, antcin C, antcin H (Zhankuic acid C), dehydrosulphurenic acid, antcin B (Zhankuic acid A), antcin A and dehydroeburicoic acid. Because the lowest IC_50_ value was observed in HCT116 cells, these cells were used in subsequent experiments.

### ACF2 upregulates *CHOP* and *TRB3* expression

**Figure 3:**
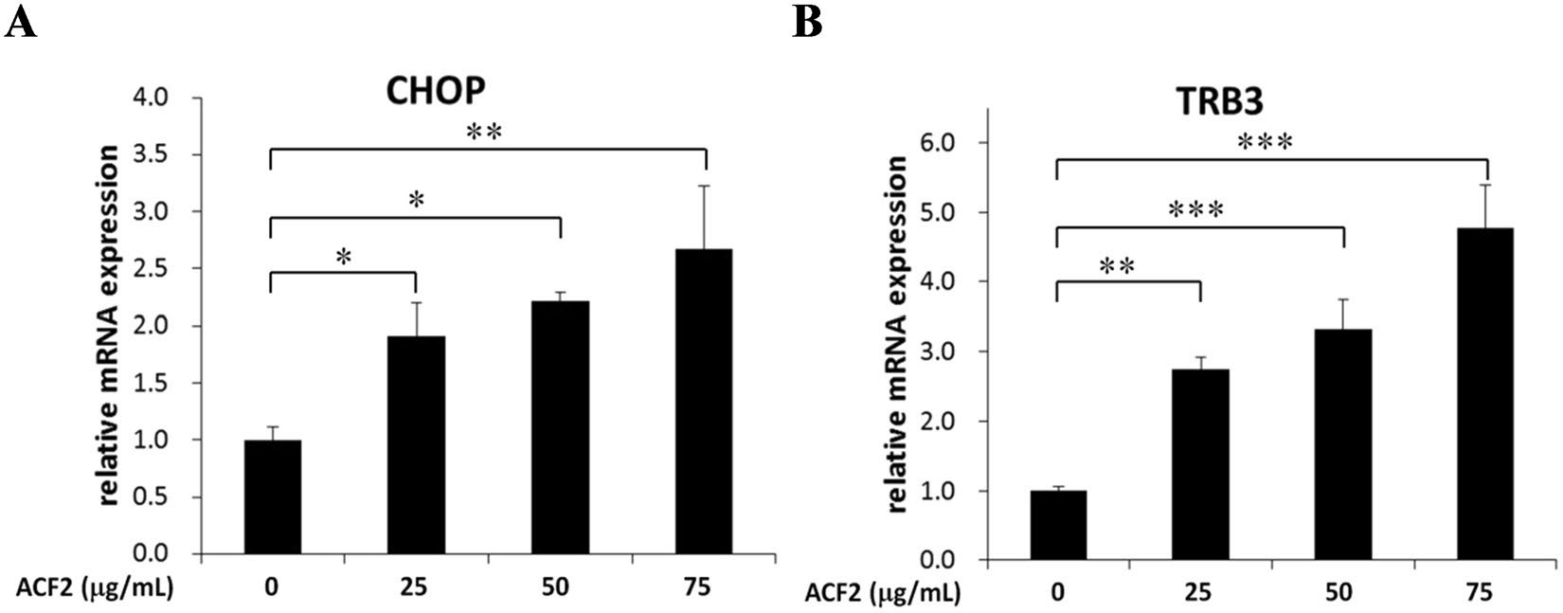
Effect of ACF2 treatment on CHOP and TRB3 mRNA expression. The levels of (**A**) CHOP and (**B**) TRB3 mRNA were analysed by real-time PCR after HCT116 cells were treated with 25, 50, and 75 μg/ml ACF2 for 24 h. ACF2 increased CHOP and TRB3 mRNA expression in a dose-dependent manner. All of the results are expressed as the mean ± standard deviation of three independent experiments. P values of statistical significance are represented as *p < 0.05, **p < 0.005 and ***p < 0.0005.

**Figure 4:**
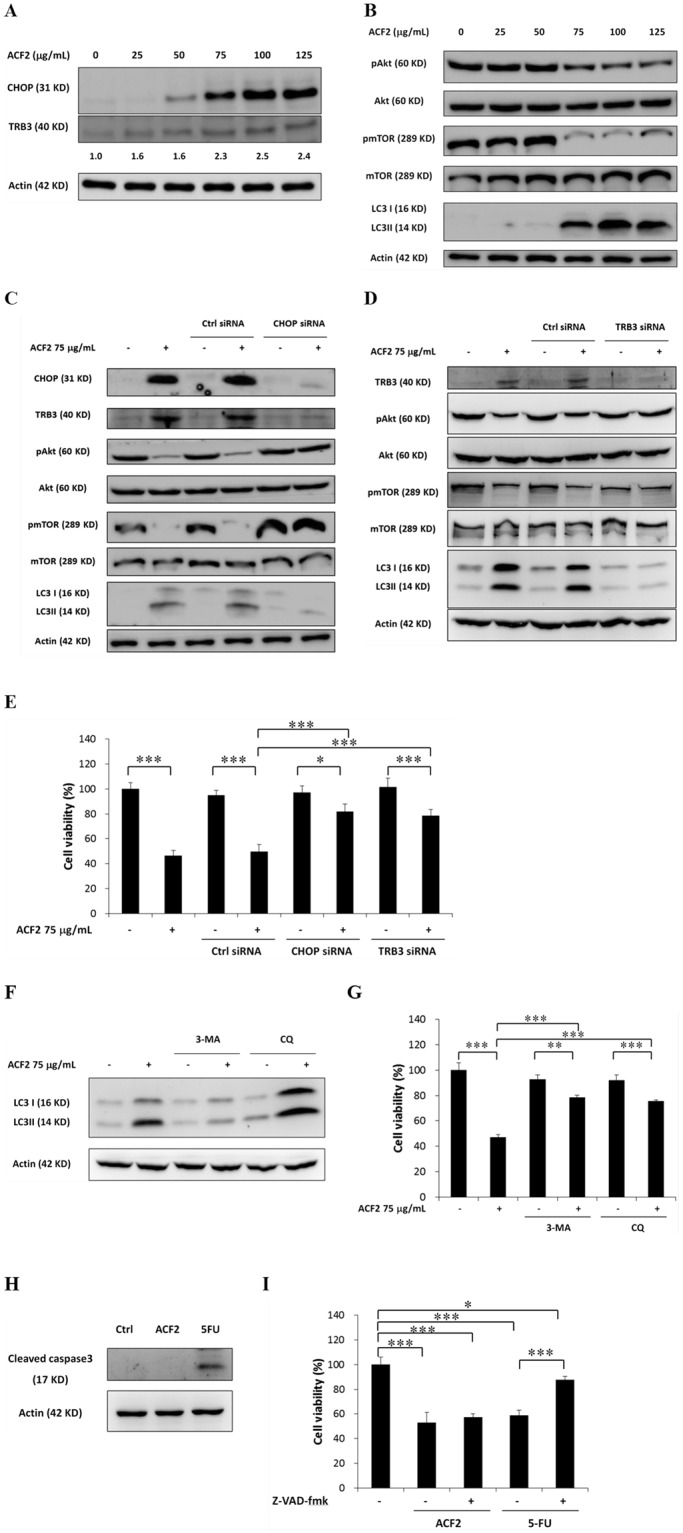
Effect of ACF2 treatment on the CHOP/TRB3/Akt/mTOR pathway and autophagic cell death. HCT116 cells were treated with 25, 50, 75, 100, and 125 μg/ml ACF2 for 24 h. The levels of (**A**) CHOP, TRB3, (**B**) total and phosphorylated Akt, total and phosphorylated mTOR, LC3, and actin protein were analysed by Western blot. Densitometry analysis of TRB3 expression was performed using ImageJ software. HCT116 cells were transfected with (**C**) CHOP siRNA, (**D**) TRB3 siRNA, or control siRNA. Non-transfected or siRNA-transfected HCT116 cells were treated with 75 μg/ml ACF2 for 24 h. Representative Western blot results of CHOP, TRB3, total and phosphorylated Akt, total and phosphorylated mTOR, LC3, and actin protein are shown. (**E**) Non-transfected and siRNA-transfected HCT116 cells were treated with 75 μg/ml ACF2 for 48 h. The cell viability was analysed by the MTS assay and expressed as cell viability (% control). HCT116 cells were treated with (**F**) 0.75 mM 3-MA, 10 μM CQ, (**H**) 100 μM 5-FU, and 75 μg/ml ACF2 for 24 h. (**F**) LC3, (**H**) cleaved caspase3, and actin protein were analysed by Western blot. HCT116 cells were treated with (**G**) 0.75 mM 3-MA, 10 μM CQ, (**H**) 100 μM 5-FU, 20 μM Z-VAD-fmk, and 75 μg/ml ACF2 for 48 h. Cell viability was analysed by the MTS assay and expressed as cell viability (% control). 5-FU was used as a positive control. Actin protein was used to normalize the results in (**A**–**D**), (**F**), and (**H**). The groups of images were cropped from different blots. Full-length blots are presented in Supplementary Figures 2 and 3. (**E**), (**G**), and (**I**) All results are expressed as the mean ± standard deviation of three independent experiments. P values of statistical significance are represented as *p < 0.05, **p < 0.005 and ***p < 0.0005.

To investigate genetic expression changes between control and ACF2-treated colorectal cancer cells, we analysed the transcriptomic profiles by NGS. Briefly, HCT116 cells were treated with 50 μg/ml ACF2 for 24 h, and RNA was then extracted from control and ACF2-treated cells. Subsequently, whole-genome sequencing was performed by NGS. A posterior probability fold change >2 and *p* < 0.05 were required for differences to be considered statistically significant. A total of 502 genes exhibited differential expression in ACF2-treated cells compared with that in control cells. Compared with control cells, 201 genes were upregulated in ACF2-treated cells, and 301 genes were downregulated (Supplementary Dataset 1). The 15 top differentially expressed genes are listed in Table 1. ACF2 significantly upregulated the expression of the endoplasmic reticulum stress (ER stress) marker C/EBP homologous protein (CHOP, also known as DNA damage-inducible transcript 3) by 12.95-fold. To investigate the anticancer mechanism of ACF2, we focused on CHOP upregulation. As a transcriptional factor, CHOP has been shown to regulate numerous apoptosis- and autophagy-related genes, such as BCL2, beclin 1, Atg5, and Atg7^26,27^. The genes listed in Table 2 are downstream genes of CHOP that are involved in apoptosis and autophagy^26–31^. After ACF2 treatment, NGS analysis showed no statistically significant difference in these genes except for tribbles pseudokinase 3 (TRIB3, also known as TRB3), which was increased by 6.48-fold after ACF2 treatment (Table 2). To confirm the NGS analysis results, quantitative reverse transcription PCR was used to observe CHOP and TRB3 gene expression. The mRNA expression levels of CHOP and TRB3 were significantly increased by ACF2 treatment, supporting the results of the NGS analysis (Fig. 3). Moreover, ACF2 increased CHOP and TRB3 mRNA expression levels in a dose-dependent manner. To investigate whether increased CHOP and TRB3 expression was associated with protein expression, Western blots were further employed. HCT116 cells were treated with multiple concentrations of ACF2 for 24 h. Western blot analysis revealed that the protein expression of CHOP was increased after treatment with ACF2 at concentrations of 50, 75, 100, and 125 μg/mL, and TRB3 was also increased by treatment with ACF2 at these concentrations. These data suggested that ACF2 markedly increases the RNA and protein expression of CHOP and TRB3 (Tables 1 and 2, Figs 3, 4A).

**Table 1:** TOP 15 differentially expressed genes between control and ACF2-treated cells according to EBSeq.

Gene	Gene ID	Gene name	PPEE	PostFC
CXCL8	3576	C-X-C motif chemokine ligand 8	0	489.24
SERF2-C15ORF63	100529067	SERF2-C15orf63 readthrough	0	254.59
TBC1D3L	101060376	TBC1 domain family member 3L	0	214.78
CXCL1	2919	C-X-C motif chemokine ligand 1	0	80.56
CXCL2	2920	C-X-C motif chemokine ligand 2	0	45.79
SERPINE1	5054	serpin family E member 1	0	21.21
CXCL3	2921	C-X-C motif chemokine ligand 3	3.55E-15	35.07
CMYA5	202333	cardiomyopathy associated 5	4.60E-14	20.54
ZFP91-CNTF	386607	ZFP91-CNTF readthrough (NMD candidate)	4.76E-13	123.96
SMIM11B	102723553	small integral membrane protein 11B	8.73E-13	0.02
DDIT3	1649	DNA damage-inducible transcript 3	1.13E-12	12.95
PRR5L	79899	proline rich 5-like	1.13E-12	48.78
GUCA1B	2979	guanylate cyclase activator 1B	1.15E-12	14.75
MTHFD2L	441024	methylenetetrahydrofolate dehydrogenase (NADP^+^ dependent) 2-like	2.85E-12	13.60
CCL20	6364	C-C motif chemokine ligand 20	4.08E-12	111.78

Gene identification second-base gene data, the National Center for Biotechnology Information (NCBI, http://www.ncbi.nlm.nih.gov/gene).

**Table 2:** Expression of CHOP and its downstream genes in control and ACF2-treated cells according to EBSeq.

Gene	Gene ID	Gene name	PPEE	PostFC
DDIT3	1649	DNA damage-inducible transcript 3	1.13E-12	12.95
TRIB3	57761	tribbles pseudokinase 3	1.21E-06	6.48
BCL2	596	BCL2, apoptosis regulator	0.98	1.13
BCL2L1	598	BCL2-like 1	0.82	2.31
BAX	581	BCL2 associated X, apoptosis regulator	0.97	1.69
BCL2L11	10018	BCL2-like 11	0.99	1.08
BBC3	27113	BCL2 binding component 3	0.91	1.91
BNIP3L	665	BCL2 interacting protein 3-like	0.95	0.56
BECN1	8678	beclin 1	0.98	0.63
CASP3	836	caspase 3	0.99	1.20
TNFRSF10B	8795	TNF receptor superfamily member 10b	0.97	1.82
PPP1R15A	23645	protein phosphatase 1 regulatory subunit 15A	0.07	3.20
ERO1A	30001	endoplasmic reticulum oxidoreductase 1 alpha	0.99	1.49
ATG5	9474	autophagy-related 5	0.99	1.14
ATG7	10533	autophagy-related 7	0.08	0.29
GABARAPL2	11345	GABA type A receptor-associated protein-like 2	0.99	1.14
ATG10	83734	autophagy-related 10	0.19	0.31
ATG12	9140	autophagy-related 12	0.98	1.59
ATG16L1	55054	autophagy-related 16-like 1	0.98	1.53
SQSTM1	8878	sequestosome 1	0.83	2.35
NBR1	4077	NBR1, autophagy cargo receptor	0.99	1.48
GABARAP	11337	GABA type A receptor-associated protein	0.99	1.49

### ACF2 induces autophagic cell death via the CHOP/TRB3/Akt/mTOR pathway

To investigate the anticancer mechanism of ACF2, we analysed molecules downstream of CHOP and TRB3 at the protein level. TRB3 is involved downstream of the CHOP-mediated apoptosis pathway^31^. TRB3 promotes apoptosis via Akt dephosphorylation, which is followed by PUMA (BBC3) induction^32,33^. However, as shown in Table 2, there was no statistically significant difference in BBC3 between control and ACF2-treated cells. We thus converted our attention to the Akt-mammalian target of rapamycin (mTOR) pathway. TRB3 reportedly negatively regulates the Akt-mTOR pathway by directly binding to Akt^34^, and negative regulation of the Akt-mTOR axis activates Ulk1. After the release of ULK1 from inactive mTOR, autophagosome formation is induced^29^. HCT116 cells were treated with increasing concentrations of ACF2 for 24 h, and phospho-Akt, total Akt, phospho-mTOR, and total mTOR were detected by Western blot analysis. As shown in Fig. 4B, at concentrations of 75, 100, and 125 μg/mL, ACF2 obviously decreased the phosphorylation levels of Akt and mTOR. The amounts of total Akt and mTOR proteins remained relatively constant regardless of whether the HCT116 cells were exposed to ACF2. Because previous studies have shown that mTOR is an important regulator of the autophagic process, autophagy was then evaluated. Microtubule-associated protein light chain 3 (LC3) is a soluble protein used to monitor autophagic activity. During the autophagic process, the soluble form of LC3 (LC3-I) is conjugated to phosphatidylethanolamine. The LC3-phosphatidylethanolamine conjugate (LC3-II) is tightly bound to autophagosomal membranes, and the LC3 conversion from LC3I to LC3II is considered one of the important hallmarks of autophagy^35^. We evaluated the autophagic process via detecting LC3 conversion by Western blot analysis. As shown in Fig. 4B, at concentrations of 75, 100, and 125 μg/mL, ACF2 obviously increased the protein expression levels of LC3-II. The accumulation of LC3-II indicated that ACF2 induced autophagy in HCT116 cells. Thus, ACF2 presumably induces autophagy via the CHOP/TRB3/Akt/mTOR pathway (Fig. 4A,B).

To verify our hypothesis that the anticancer mechanism of ACF2 in colorectal cancer is due to CHOP/TRB3/Akt/mTOR-mediated autophagy, CHOP and TRB3 small interference RNA (siRNA) were employed to suppress CHOP and TRB3 expression in HCT116 cells. HCT116 cells were transfected with siRNA targeting human CHOP or TRB3 or control siRNA. After 48 h of stimulation, HCT116 cells were treated with 75 μg/mL ACF2 for 24 h. Compared with non-transfected and control siRNA-transfected cells, CHOP siRNA clearly decreased ACF2-induced CHOP protein overexpression (Fig. 4C). TRB3 siRNA decreased ACF2-induced TRB3 protein overexpression (Fig. 4D). Phospho-Akt, total Akt, phospho-mTOR, and total mTOR were also detected by Western blot analysis. As shown in Fig. 4C, the CHOP protein reduction was accompanied by efficient reduction of ACF2-induced TRB3 protein expression in CHOP siRNA-transfected cells. Moreover, CHOP and TRB3 siRNA significantly reversed the ACF2-decreased phosphorylation levels of Akt and mTOR. In CHOP or TRB3 siRNA-transfected cells, ACF2 treatment did not suppress Akt or mTOR phosphorylation. Next, we evaluated LC3-II protein expression in CHOP or TRB3 siRNA-transfected cells, revealing that both CHOP and TRB3 knockdown decreased ACF2-induced LC3-II expression (Fig. 4C,D).

Since autophagy has been implicated in cell survival and cell death^29^, we next investigated the role of autophagy in the anticancer mechanism of ACF2. The MTS assay was used to evaluate the viability of HCT116 cells. As shown in Fig. 4E, transfection with control siRNA or TRB3 or CHOP siRNA had no effect on cell viability. Compared with non-transfected and control siRNA-transfected cells treated with ACF2, in the presence of CHOP or TRB3 siRNA, ACF2-induced cell death was significantly diminished. Next, 3-methyladenine (3-MA) and chloroquine (CQ), autophagy inhibitors, were employed to inhibit autophagy. LC3-II protein expression and the cell viability of HCT116 cells were evaluated. As shown in Fig. 4F, ACF2-induced LC3-II protein increase was efficiently reduced by 3-MA but increased by CQ. In the presence of 3-MA or CQ, ACF2-induced cell death was significantly decreased (Fig. 4G). To exclude apoptotic cell death in this study, cleaved caspase3 protein expression and the cell viability of HCT116 cells were assessed. 5-Fluouracil (5-FU) was used as a positive control to induce apoptosis in HCT116 cells^36^. 5-FU obviously increased the protein expression levels of cleaved caspase3. Cleaved caspase3 was not observed after ACF2 treatment (Fig. 4H). Z-VAD-fmk, a pan-caspase inhibitor, was employed to inhibit apoptosis. As shown in Fig. 4I, in the presence of Z-VAD-fmk, 5-FU-induced apoptotic cell death was significantly diminished. However, Z-VAD-fmk did not rescue ACF2-induced cell death. ACF2 treatment can lead to autophagy-dependent cell death. Taken together, these results suggest that ACF2 induces autophagic cell death via the CHOP/TRB3/Akt/mTOR pathway.

### ACF2 inhibits the growth of colorectal cancer cells *in vivo*

**Figure 5:**
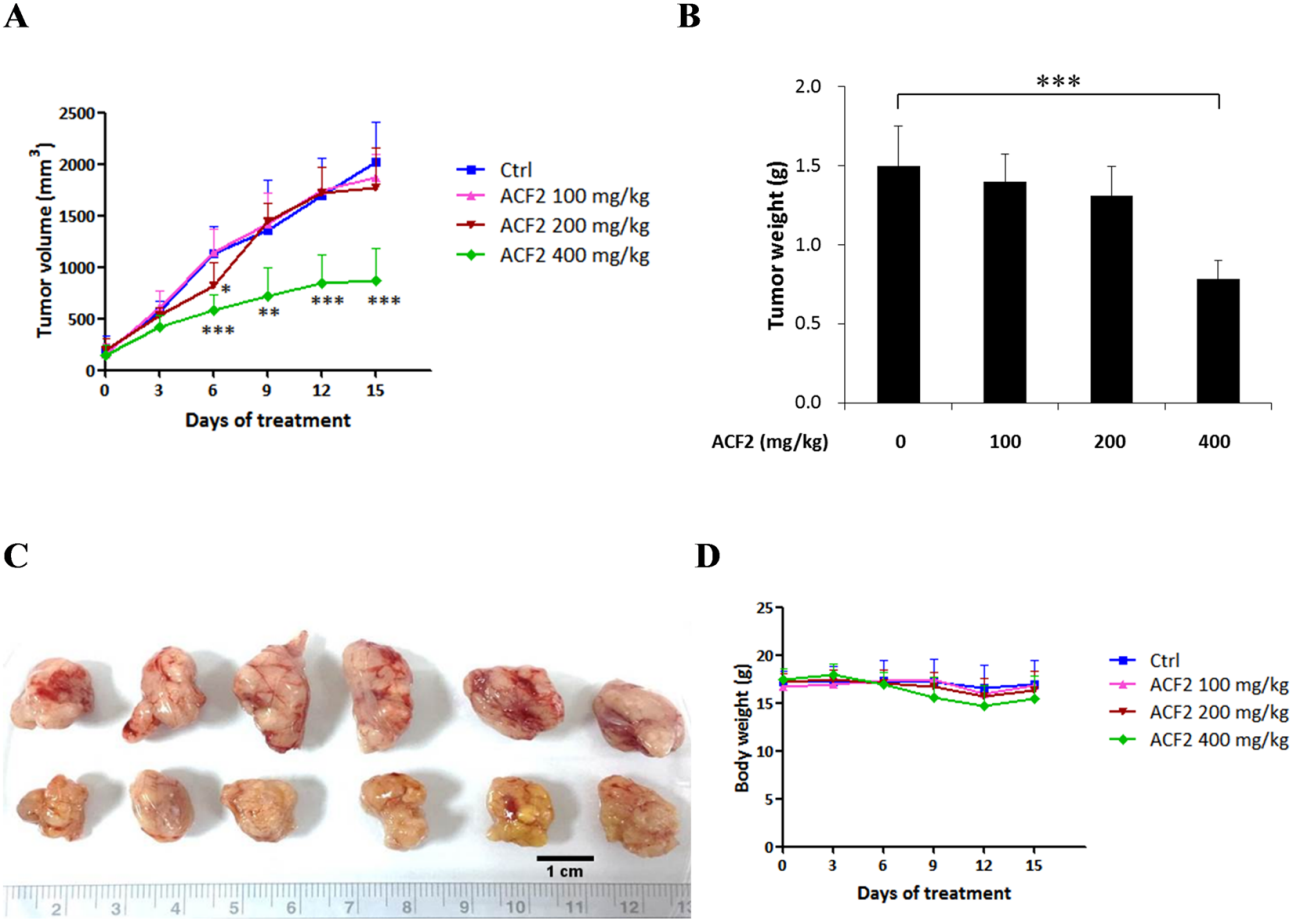
Effect of ACF2 treatment on tumour volume, tumour weight, and body weight in human HCT116 xenograft tumour-bearing mice. On the seventh day after tumour inoculation, the effects of ACF2 were evaluated. Animals were divided into four groups, including the control group (treated with 0.2 mL of normal saline) and the 100, 200, and 400 mg/kg ACF2 treatment groups. (**A**) Average tumour volume. (**B**) Average tumour weight. (**C**) Representative subcutaneous tumours in the control and 400 mg/kg ACF2 treatment groups. (**D**) Average body weight. All results are expressed as the mean ± standard deviation; N = 6 for all groups. P values of statistical significance are represented as *p < 0.05 and ***p < 0.0005.

To investigate whether ACF2 has an anticancer effect on colorectal cancer *in vivo*, athymic mice xenografted with a human tumour model were utilized. HCT116 cells were inoculated into the nude mice at 3 × 10^6^ cells per mouse subcutaneously and treated with ACF2 at doses of 400 mg/kg, 200 mg/kg, and 100 mg/kg (daily gavage) for 15 days. As shown in Fig. 5A, the tumour volume in the control group (Ctrl) increased gradually from days 0 to 15 after administration. From day 6 onwards, the tumour volume in the 400 mg/kg ACF2 treatment group increased slowly, and the differences between the control group and the 400 mg/kg ACF2 treatment group became significant (*p* < 0.005). On the sixth day after treatment, the tumour volume in the 200 mg/kg ACF2 treatment group was significantly decreased compared with that in the control group (*p* < 0.05). However, after 9, 12 and 15 days of administration, the tumour volume in the 200 mg/kg ACF2 treatment group increased, and there was no statistically significant difference compared with the control group. Changes in tumour volume in the 100 mg/kg ACF2 treatment group were similar to those in the control group, with the two groups presenting no significant difference at any time point. As shown in Fig. 5B,C, at the end of the 15-day treatment period, the average weight of the tumours in the 400 mg/kg ACF2 treatment group was significantly smaller than that in the control group (*p* < 0.005). The body weight of each mouse was observed during the experiments. Importantly, there were no significant decreases in body weight in the ACF2 treatment groups at any of the dosages (Fig. 5D). These results indicate that ACF2 can inhibit the growth of HCT116 colorectal cancer cells *in vivo*.

## Discussion

In this study, we utilized whole-genome expression profiling to discover the molecular mechanisms underlying the anticancer effects of *A. cinnamomea* on colorectal cancer. In previous studies, *A. cinnamomea* was determined to exert anticancer effects on colorectal cancer by the induction of apoptotic cell death and cell cycle arrest^10,17,19,20^. However, the upstream molecular mechanisms of these anticancer effects remained unknown. We used NGS to uncover novel mechanisms of *A. cinnamomea*, revealing that the *A. cinnamomea* treatment of HCT116 colorectal cancer cells resulted in the upregulation of CHOP, TRB3, and some members of the CXC chemokine family.

CHOP, known as growth arrest and DNA damage 153 (GADD153), was originally identified in response to UV-induced DNA damage^37^. However, further studies showed that ER stress plays a critical role in CHOP induction. During ER stress, CHOP was induced by all three ER stress sensors, namely, PERK, IRE1, and ATF6^28–31,38^. The numerous inducers of CHOP, such as DNA-alkylating agent methylmethane sulphate, tunicamycin, and thapsigargin, activated CHOP expression via the induction of ER stress^38–40^. *A. cinnamomea* reportedly induces ER stress-mediated apoptosis and autophagy in lung and liver cancer cells^41,42^. In our study, *A. cinnamomea* treatment induced CHOP-mediated autophagic cell death in colorectal cancer cells.

After CHOP induction, CHOP promotes the transcriptional activation of many pro-apoptotic factors, including TNFRSF10B, ERO1A, TRB3, and members of the BCL2 family. CHOP-mediated apoptosis is involved in many diseases, such as neurodegenerative diseases, metabolic diseases, atherosclerosis, and cancer^31^. CHOP has been shown to trigger apoptotic cell death in lung, colorectal, prostate, and ovarian cancer^43,44^. On the other hand, CHOP also induces the transcriptional upregulation of many autophagic genes, such as SQSTM1, NBR1, and members of the ATG family^27^. In our study, the expression of CHOP and its downstream target TRB3 were upregulated by *A. cinnamomea*. TRB3 can promote apoptosis via Akt dephosphorylation, followed by BBC3 induction^32,33^. TRB3 can also promote autophagy via Akt and mTOR dephosphorylation^29^. Akt and mTOR dephosphorylation were observed after *A. cinnamomea* treatment, but the mRNA expression of BBC3 was not increased. Conversion of the autophagy marker LC3 was increased by *A. cinnamomea* treatment (Table 2 and Fig. 4B). Furthermore, knockdown of CHOP and TRB3 prevented the effects of *A. cinnamomea* on TRB3, Akt, mTOR, and LC3 expression (Fig. 4C,D) and diminished *A. cinnamomea*-induced cell death (Fig. 4E). Autophagy inhibition but not apoptosis inhibition reversed *A. cinnamomea*-induced cell death (Fig. 4G,I). These findings reveal that autophagic cell death is induced via the CHOP/TRB3/Akt/mTOR pathway in response to *A. cinnamomea* treatment. Apoptotic cell death was observed in colorectal, lung and liver cancer cells after *A. cinnamomea* treatment^10,17,42,45^. On the other hand, *A. cinnamomea* induced autophagic cell death in liver, pancreatic, head and neck cancer cells^41,46,47^. Our study identified that *A. cinnamomea* also induced autophagic cell death in colorectal cancer.

Chemokines are involved in many cellular processes, such as inflammatory, innate and adaptive responses; tumour growth; and tumour angiogenesis^48–51^. CXCL1, CXCL2, CXCL3, and CXCL8 are members of CXC chemokine family. CXCL8 has been reported as a central gene that regulates a colon cancer network^52^. High correlation was observed between CXCL8 and other cytokines (CXCL1, CXCL2, and CXCL3)^53^. Previous studies showed that CXCL8 promoted cell proliferation via the activation of Akt and inhibited apoptosis in colorectal cancer^54–56^. In response to the chemotherapy drug 5-fluorouracil, HCT116 cells upregulated the expression of CXCL8, CXCR1, and CXCR2; CXCR1 and CXCR2 are receptors of CXCL8^57^. In our study, CXCL1, CXCL2, CXCL3, and CXCL8 were upregulated after *A. cinnamomea* treatment. However, our results indicated that *A. cinnamomea* has anticancer effects both *in vitro* and *in vivo* (Figs 2, 5). As shown in Fig. 4, *A. cinnamomea* induced autophagic cell death and inhibited the phosphorylation of Akt. The molecular pathway of *A. cinnamomea* is different from that of CXCL8. We assumed that upregulation of CXC family members is a cellular response after *A. cinnamomea* treatment. *A. cinnamomea* exerted excellent anticancer effects on colorectal cancer.

The whole-genome expression profiling of *A. cinnamomea* in colorectal cancer was established for the first time. We clearly demonstrated the anticancer molecular mechanisms of *A. cinnamomea* in colorectal cancer, revealing that *A. cinnamomea* induces autophagic cell death via the CHOP/TRB3/Akt/mTOR pathway. These findings provide a reference for future *A. cinnamomea* drug development for colorectal cancer treatment.

## Methods

### Preparation of *A. cinnamomea* fruiting body extracts

*A. cinnamomea* (Fig. 1A) was cultured on dishes for four months by Yong Teng Biotechnology Co., Ltd. (New Taipei City, Taiwan). The *A. cinnamomea* strain was confirmed by the PCR fragments of the internally transcribed spacers of the ribosomal RNA genes. The *A. cinnamomea* fruiting bodies (approximately 100 g) were soaked in 3000 mL of ethanol for 120 h. The samples were extracted three times using filter paper (Advantec No. 1, Tokyo, Japan). The combined filtrates collected from the three extractions were evaporated to dryness under vacuum. The ethanol extract (23 g, AC) from the powdered fruiting bodies of *A. cinnamomea* (100 g) was applied to a Diaion HP-20 column (460 g) and washed with 50% ethanol, 95% ethanol, and ethyl acetate to yield 3 fractions: ACF1 (14.71 g, yield [w/w] 71.32%), ACF2 (6.11 g, yield [w/w] 29.64%), and ACF3 (0.75 g, yield [w/w] 3.65%). The scheme depicting the methodology is shown in Fig. 1B.

### Cell culture

Five human colorectal cancer cell lines (HCT116, HT29, SW480, Caco-2, and Colo205) were obtained from the Bioresource Collection and Research Center (Hsinchu, Taiwan). Dulbecco’s Modified Eagle’s Medium containing 10% foetal bovine serum, 100 U/ml penicillin and 0.1 mg/ml streptomycin served as the cell culture medium throughout all experiments. The cells were maintained at 37 °C in a humidified atmosphere of 5% CO_2_ in air and passaged every 2 days to obtain exponential growth. All five cell lines were maintained in culture no more than 15 passages. All culture reagents were sourced from Gibco (Invitrogen, Carlsbad, CA, USA).

### MTS assay

HCT116, HT29, SW480, Caco-2, and Colo205 cells were seeded in 96-well plates at a density of 5 × 10^4^ cells per well and treated with increasing concentrations of AC, ACF1, ACF2 or ACF3 for 48 h. The cytotoxic effects of AC, ACF1, ACF2, and ACF3 on colorectal cancer cells were assessed by the MTS assay (Promega, Fitchburg, WI, USA). The absorbance was measured at 490 nm with a microplate reader. The IC_50_ values were calculated using GraphPad Prism 5.

### HPLC analysis

ACF2 was further separated using a Thermo HPLC system (Thermo Fisher Scientific, Waltham, MA, USA) with an Eclipse XDB-C18 analytical column (Agilent Technologies, Santa Clara, CA, USA). Gradient elution was performed with mobile phases A (0.1% formic acid aqueous solution) and B (acetonitrile). The gradient elution profile was as follows: 0–3 minutes, 70% A and 30% B to 60% A and 40% B (linear gradient); 3–15 minutes, 60% A and 40% B to 42% A and 58% B (linear gradient); 15–21 minutes, 42% A and 58% B (isocratic); 21–26 minutes, 42% A and 58% B to 35% A and 65% B (linear gradient); 26–35 minutes, 35% A and 65% B to 0% A and 100% B (linear gradient); and 35–50 minutes, 0% A and 100% B (isocratic). The flow rate was 0.8 mL/min, and the wavelength of the photodiode array detector was 254 nm.

### NGS analysis

HCT116 cells were seeded in 10 cm plates at a density of 2 × 10^6^ cells per plate and treated with 50 μg/ml ACF2 for 24 h. Total RNA was extracted using the RNeasy Mini Kit (Qiagen, Hilden, Germany).

An RNA sequencing library was generated using the TruSeq Stranded mRNA Library Prep Kit according to the user’s instruction manual (Illumina, San Diego, CA, USA). Briefly, the poly-A containing mRNA was isolated from total RNA using poly-T oligo-attached magnetic beads and fragmented and primed for cDNA synthesis. After double-strand cDNA synthesis, adenylation of the 3′-end, and multiple indexing adapter ligation, DNA purification with magnetic beads and PCR selective amplification were performed. Next, the amplified library was purified, quantified, and applied for template preparation. The NextSeq 500 platform was utilized to generate 75-bp single-end sequencing reads. The initial sequence reads were generated by the Genome Analyser (Illumina, San Diego, CA, USA), and the expression values were calculated using RNA-Seq by Expectation Maximization (RSEM) for each gene (University of California, Santa Cruz (UCSC), Homo.sapiens.hg19.gtf) in individual samples^58^. A total of 20 million reads were randomly selected for quality control with Bowtie 2 and mapped to Genome Reference Consortium Human Build 37/hg19^59^. Differential expression was analysed between different subgroups using Bioconductor EBseq. (version 1.1.6)^60^. A false discovery rate of 0.05 was applied to the resulting P values to correct for multiple hypothesis testing. T-tests were employed to screen genes differentially expressed between control and ACF2-treated cells, with a threshold of P < 0.05 and a posterior probability fold change >2.

### Quantitative reverse transcription PCR

HCT116 cells were treated with 25, 50, and 75 μg/ml ACF2 for 24 h. Total RNA was isolated using the RNeasy Mini Kit (Qiagen, Hilden, Germany). cDNA was synthesized from the RNA using the SuperScript III First-Strand Synthesis System (Invitrogen, Carlsbad, CA, USA) according to the manufacturer’s instructions. Quantitative real-time PCR was performed using iQ SYBR Green Supermix (Bio-Rad, Hercules, CA, USA) with the following specific primers: CHOP (forward primer, 5′-CAG AAC CAG AGG TCA CA-3′; reverse primer, 5′-AGC TGT GCC ACT TTC CTT TC-3′)^61^, TRB3 (forward primer, 5′-TGG TAC CCA GCT CCT CTA CG-3′; reverse primer, 5′-GAC AAA GCG ACA CAG CTT GA-3′), and GAPDH (forward primer, 5′-GAA GGT GAA GGT CGG AGT C-3′; reverse primer, 5′-GAA GAT GGT GAT GGG ATT TC-3′)^62^. Reactions were performed using the CFX Connect Real-Time PCR Detection System (Bio-Rad, Hercules, CA, USA) with the following reaction programme: 95 °C for 10 min, followed by 40 cycles of 95 °C for 15 s and 60 °C for 1 min. The relative changes in gene expression were calculated by the 2^−ΔΔCT^ method, and GAPDH served as the internal control.

### Western blot analysis

HCT116 cells were treated with 25, 50, 75, 100, and 125 μg/ml ACF2 for 24 h. Total cellular protein was extracted using M-PER™ mammalian protein extraction reagent (Thermo Fisher Scientific, Waltham, MA, USA) and then quantified using the Bradford protein assay (Bio-Rad, Hercules, CA, USA). Denatured protein was loaded onto 10–15% SDS polyacrylamide gels. Following electrophoresis, the proteins on the gel were transferred onto polyvinylidene difluoride membranes using the TE22 mighty small transfer tank (Hoefer, Holliston, MA, USA). The membranes were blocked with 5% skim milk at room temperature for 30 minutes, followed by incubation at 4 °C overnight with the following primary antibodies: CHOP, TRB3, caspase3, actin (Abcam, Milton, Cambridge, UK), total and phosphorylated Akt (ser473), total and phosphorylated mTOR (ser2448), and LC3 (Cell Signaling Technology, Danvers, MA, USA). The membranes were then washed three times for 10 minutes with PBS-Tween buffer and incubated with horseradish peroxidase-conjugated anti-mouse or anti-rabbit secondary antibodies (Abcam, Milton, Cambridge, UK) at room temperature for 1 h. All antibodies were diluted 1:2,000. The antibody binding proteins were detected using ECL chemiluminescence substrates and then captured on the Amersham Imager 600 (GE Healthcare, Chicago, IL, USA). The membranes were reprobed for actin protein expression to show that similar amounts of protein were loaded in each lane.

### siRNA transfection

For siRNA inhibition, double-stranded RNA duplexes targeting human CHOP (5′- GCC UGG UAU GAG GAC CUG C-3′) or human TRB3 (5′-CGA GCU CGA AGU GGG CCC C-3′) and control siRNA (5′-GCG CGC UUU GUA GGA UUC G-3′)^32^ were synthesized by Invitrogen. HCT116 cells were transfected with siRNA using Lipofectamine RNAiMAX (Invitrogen, Carlsbad, CA, USA) according to the manufacturer’s protocol.

### *In vivo* experiments

Six-week-old male nude^nu/nu^ mice were obtained from the National Laboratory Animal Center (Taipei, Taiwan) and housed in clean specific pathogen-free (SPF) rooms. The mice were inoculated with 3 × 10^6^ HCT116 cells suspended in PBS subcutaneously into the right flanks. Twenty-four mice were randomly divided into four groups (6 mice in each group): a control group (treated with 0.2 mL of normal saline) and 100, 200, and 400 mg/kg ACF2 treatment groups. ACF2 treatment was started on the seventh day after tumour inoculation by daily oral gavage for 15 days. The body weights of the mice were recorded every three days. Tumour volume was measured every three days using digital callipers and calculated as length × (width)^2^/2. The experimental protocol was approved by the Institutional Animal Care and Use Committee at National Taiwan University. All methods were carried out in accordance with relevant guidelines and regulations.

### Statistical analysis

All experiments were performed in triplicate, and data are presented as the mean ± standard deviation. Statistical analysis was performed by the ANOVA and LSD tests using SPSS 12.0 software (SPSS, Inc., Chicago, IL, USA). P values of statistical significance are represented as *p < 0.05, **p < 0.005 and ***p < 0.0005.

## Electronic supplementary material

Supplementary file

Dataset 1

## References

[1] Ferlay J (2015). Cancer incidence and mortality worldwide: sources, methods and major patterns in GLOBOCAN 2012. Int. J. Cancer.

[2] Torre LA, Siegel RL, Ward EM, Jemal A (2016). Global cancer incidence and mortality rates and trends–an update. Cancer Epidemiol. Biomarkers Prev..

[3] Demain AL, Vaishnav P (2011). Natural products for cancer chemotherapy. Microb. Biotechnol..

[4] Mann John (2002). Natural products in cancer chemotherapy: past, present and future. Nature Reviews Cancer.

[5] Mitra S, Dash R (2018). Natural products for the management and prevention of breast cancer. Evid. Based Complement. Alternat. Med..

[6] Geethangili M, Tzeng YM (2011). Review of Pharmacological Effects of Antrodia camphorata and Its Bioactive Compounds. Evid. Based Complement. Alternat. Med..

[7] Tsai, Z. T. & Liaw, S. L. *The use and the effect of ganoderma*. 116Taiwan (San Yun Press, 1985).

[8] Huang GJ (2010). Analgesic effects and the mechanisms of anti-inflammation of ergostatrien-3beta-ol from Antrodia camphorata submerged whole broth in mice. J. Agric. Food Chem..

[9] Hsu YL (2007). Antrodia cinnamomea fruiting bodies extract suppresses the invasive potential of human liver cancer cell line PLC/PRF/5 through inhibition of nuclear factor kappaB pathway. Food Chem. Toxicol..

[10] Lien HM (2011). Study of the Anti-Proliferative Activity of 5-Substituted 4,7-Dimethoxy-1,3-Benzodioxole Derivatives of SY-1 from Antrodia camphorata on Human COLO 205 ColonCancer Cells. Evid. Based Complement. Alternat. Med..

[11] Wu H (2006). Proteomic analysis of the effect of Antrodia camphorata extract on human lung cancer A549 cell. Proteomics.

[12] Hseu YC (2007). Inhibition of cyclooxygenase-2 and induction of apoptosis in estrogen-nonresponsive breast cancer cells by Antrodia camphorata. Food Chem. Toxicol..

[13] Lu MC (2009). Active extracts of wild fruiting bodies of Antrodia camphorata (EEAC) induce leukemia HL 60 cells apoptosis partially through histone hypoacetylation and synergistically promote anticancer effect of trichostatin A. Arch. Toxicol..

[14] Kuo MC, Chang CY, Cheng TL, Wu MJ (2008). Immunomodulatory effect of Antrodia camphorata mycelia and culture filtrate. J. Ethnopharmacol..

[15] Song Tuzz-Ying, Yen Gow-Chin (2003). Protective Effects of Fermented Filtrate fromAntrodia camphoratain Submerged Culture against CCl4-Induced Hepatic Toxicity in Rats. Journal of Agricultural and Food Chemistry.

[16] Hsiao G (2003). Antioxidative and hepatoprotective effects of Antrodia camphorata extract. J. Agric. Food Chem..

[17] Park DK, Lim YH, Park HJ (2013). Antrodia camphorata grown on germinated brown rice inhibits HT-29 human colon carcinoma proliferation through inducing G0/G1 phase arrest and apoptosis by targeting the beta-catenin signaling. J. Med. Food.

[18] Lin HC (2017). Antroquinonol, a ubiquinone derivative from the mushroom antrodia camphorata, inhibits colon cancer stem cell-like properties: Insights into the molecular mechanism and inhibitory targets. J. Agric. Food Chem..

[19] Tu SH (2012). *In vivo* antitumor effects of 4,7-dimethoxy-5-methyl-1,3-benzodioxole isolated from the fruiting body of Antrodia camphorata through activation of the p53-mediated p27/Kip1 signaling pathway. J. Agric. Food Chem..

[20] Chen LY (2011). Pretreatment with an ethanolic extract of Taiwanofungus camphoratus (Antrodia camphorata) enhances the cytotoxic effects of amphotericin B. J. Agric. Food Chem..

[21] Huang L (2013). Integrating transcriptional profiling with network-based methodologies for revealing molecular mechanisms of traditional Chinese medicine. OA Alternat. Med..

[22] Li CC, Lo HY, Hsiang CY, Ho TY (2012). DNA microarray analysis as a tool to investigate the therapeutic mechanisms and drug development of Chinese medicinal herbs. Biomedicine.

[23] Wen Z (2011). Discovery of molecular mechanisms of traditional Chinese medicinal formula Si-Wu-Tang using gene expression microarray and connectivity map. PLoS One.

[24] Pan-Hammarstrom Q, Wen S, Hammarstrom L (2006). Cytokine gene expression profiles in human lymphocytes induced by a formula of traditional Chinese medicine, vigconic VI-28. J. Interferon. Cytokine Res..

[25] Sheu F (2009). Purification, cloning, and functional characterization of a novel immunomodulatory protein from Antrodia camphorata (bitter mushroom) that exhibits TLR2-dependent NF-kappaB activation and M1 polarization within murine macrophages. J. Agric. Food Chem..

[26] Fribley A, Zhang K, Kaufman RJ (2009). Regulation of apoptosis by the unfolded protein response. Methods Mol. Biol..

[27] B’Chir W (2013). The eIF2alpha/ATF4 pathway is essential for stress-induced autophagy gene expression. Nucleic Acids Res..

[28] Jing G, Wang JJ, Zhang SX (2012). ER stress and apoptosis: a new mechanism for retinal cell death. Exp. Diabetes Res..

[29] Rashid HO, Yadav RK, Kim HR, Chae HJ (2015). ER stress: Autophagy induction, inhibition and selection. Autophagy.

[30] Verfaillie T, Salazar M, Velasco G, Agostinis P (2010). Linking ER stress to autophagy: Potential implications for cancer therapy. Int. J. Cell. Biol..

[31] Li Y, Guo Y, Tang J, Jiang J, Chen Z (2014). New insights into the roles of CHOP-induced apoptosis in ERstress. Acta Biochim. Biophys. Sin. (Shanghai).

[32] Ohoka N, Yoshii S, Hattori T, Onozaki K, Hayashi H (2005). TRB3, a novel ER stress-inducible gene, is induced via ATF4-CHOP pathway and is involved in cell death. EMBO J..

[33] Zou CG (2009). The molecular mechanism of endoplasmic reticulum stress-induced apoptosis in PC-12 neuronal cells: the protective effect of insulin-like growth factor I. Endocrinology.

[34] Salazar M (2009). Cannabinoid action induces autophagy-mediated cell death through stimulation of ER stress in human glioma cells. J. Clin. Invest..

[35] Hansen TE, Johansen T (2011). Following autophagy step by step. BMC Biol..

[36] Garcia MA (2011). The chemotherapeutic drug 5-fluorouracil promotes PKR-mediated apoptosis in a p53-independent manner in colon and breast cancer cells. PLoS One.

[37] Gujuluva CN, Baek JH, Shin KH, Cherrick HM, Park NH (1994). Effect of UV-irradiation on cell cycle, viability and the expression ofp53, gadd153 and gadd45 genes in normal and HPV-immortalized human oral keratinocytes. Oncogene.

[38] Zinszner H (1998). CHOP is implicated in programmed cell death in response to impaired function of the endoplasmic reticulum. Genes Dev..

[39] Ron D, Habener JF (1992). CHOP, a novel developmentally regulated nuclear protein that dimerizes with transcription factors C/EBP and LAP and functions as a dominant-negative inhibitor of gene transcription. Genes Dev..

[40] Marciniak SJ (2004). CHOP induces death by promoting protein synthesis and oxidation in the stressed endoplasmic reticulum. Genes Dev..

[41] Su YC (2012). Eburicoic acid, an active triterpenoid from the fruiting bodies of basswood cultivated antrodia cinnamomea, induces er stress-mediated autophagy in human hepatoma cells. J. Tradit. Complement. Med..

[42] Chan YY, Chang CS, Chien LH, Wu TF (2010). Apoptotic effects of a high performance liquid chromatography (HPLC) fraction of Antrodia camphorata mycelia are mediated by down-regulation of the expressions of four tumor-related genes in human non-small cell lung carcinoma A549 cell. J. Ethnopharmacol..

[43] Yamaguchi H, Wang HG (2004). CHOP is involved in endoplasmic reticulum stress-induced apoptosis by enhancing DR5 expression in human carcinoma cells. J. Biol. Chem..

[44] Hsin IL (2012). Lipocalin 2, a new GADD153 target gene, as an apoptosis inducer of endoplasmic reticulum stress in lung cancer cells. Toxicol. Appl. Pharmacol..

[45] Lai CI (2016). Antcin K, an active triterpenoid from the fruiting bodies of basswood cultivated Antrodia cinnamomea, induces mitochondria and endoplasmic reticulum stress-mediated apoptosis in human hepatoma cells. J. Tradit. Complement. Med..

[46] Yu CC (2012). Antroquinonol, a natural ubiquinone derivative, induces a cross talk between apoptosis, autophagy and senescence in human pancreatic carcinoma cells. J. Nutr. Biochem..

[47] Chang CW (2013). Active component of antrodia cinnamomea mycelia targeting head and neck cancer initiating cells through exaggerated autophagic cell death. Evid. Based Complement. Alternat. Med..

[48] Esche C, Stellato C, Beck LA (2005). Chemokines: key players in innate and adaptive immunity. J. Invest. Dermatol..

[49] Itatani Y (2016). The role of chemokines in promoting colorectal cancer invasion/metastasis. Int. J. Mol. Sci..

[50] Wang D, Dubois RN, Richmond A (2009). The role of chemokines in intestinal inflammation and cancer. Curr. Opin. Pharmacol..

[51] Sarvaiya PJ, Guo D, Ulasov I, Gabikian P, Lesniak MS (2013). Chemokines in tumor progression and metastasis. Oncotarget.

[52] Jiang W (2008). Constructing disease-specific gene networks using pair-wise relevance metric: application to colon cancer identifies interleukin 8, desmin and enolase 1 as the central elements. BMC Syst. Biol..

[53] Ha H, Debnath B, Neamati N (2017). Role of the CXCL8-CXCR1/2 Axis in cancer and inflammatory diseases. Theranostics.

[54] Xiao YC (2015). CXCL8, overexpressed in colorectal cancer, enhances the resistance of colorectal cancer cells to anoikis. Cancer Lett..

[55] Sturm A (2005). CXCL8 modulates human intestinal epithelial cells through a CXCR1 dependent pathway. Cytokine.

[56] Itoh Y (2005). IL-8 promotes cell proliferation and migration through metalloproteinase-cleavage proHB-EGF in human colon carcinoma cells. Cytokine.

[57] Dabkeviciene D (2015). The role of interleukin-8 (CXCL8) and CXCR2 in acquired chemoresistance of human colorectal carcinoma cells HCT116. Med. Oncol..

[58] Li B, Dewey CN (2011). RSEM: accurate transcript quantification from RNA-Seq data with or without a reference genome. BMC Bioinformat..

[59] Langmead B, Salzberg SL (2012). Fast gapped-read alignment with Bowtie 2. Nat. Methods.

[60] Robinson MD, McCarthy DJ, Smyth G (2010). K. edgeR: a Bioconductor package for differential expression analysis of digital gene expression data. Bioinformatics.

[61] Averous J (2004). Induction of CHOP expression by amino acid limitation requires both ATF4 expression and ATF2 phosphorylation. J. Biol. Chem..

[62] Yan Weihui, Wang Ying, Xiao Yongtao, Wen Jie, Wu Jiang, Du Lei, Cai Wei (2014). Palmitate Induces TRB3 Expression and Promotes Apoptosis in Human Liver Cells. Cellular Physiology and Biochemistry.

