# Supplementary material for: Antrodia cinnamomea induces autophagic cell death via the CHOP/TRB3/Akt/mTOR pathway in colorectal cancer cells
Source: Sci Rep. 2018 Nov 27;8:17424. doi: 10.1038/s41598-018-35780-y (PMC6258711; doi:10.1038/s41598-018-35780-y)
Supplement: Supplementary file 1 — Supplementary file [file 41598_2018_35780_MOESM1_ESM.docx]

***Antrodia cinnamomea* induces autophagic cell death via the CHOP/TRB3/Akt/mTOR pathway in colorectal cancer cells**

# Dai-Hua Tsai^1^, Cheng-Han Chung^2^, and Kung-Ta Lee^1,*^

^1^Department of Biochemical Science and Technology, National Taiwan University, Taipei, 10617, Taiwan

^2^Yong Teng biotechnology Co., Ltd., New Taipei City, 22180, Taiwan

*ktlee@ntu.edu.tw

**Supplementary Figure 1** Effect of *A. cinnamomea* extract treatment on the cell viability of HCT116 colorectal cancer cells. HCT116 cells were treated with AC, ACF1, ACF2, and ACF3 for 48 h. The cell viability was analysed by direct trypan blue exclusion cell count and expressed as cell viability (% control). All results are expressed as the mean ± standard deviation of three independent experiments. P values of statistical significance are represented as **p* < 0.05, ** *p* < 0.005 and *** *p* < 0.0005.

**Supplementary Figure 2** Full-length blots of Figure 4A-4C. (A) CHOP, (B) TRB3, and (C) actin in Figure 4A. (D) phosphorylated Akt, (E) total Akt, (F) phosphorylated mTOR, (G) total mTOR, (H) LC3, and (I) actin in Figure 4B. (J) CHOP, (K) TRB3, (L) phosphorylated Akt, (M) total Akt, (N) phosphorylated mTOR, (O) total mTOR, (P) LC3, and (Q) actin in Figure 4C.

**Supplementary Figure 3** Full-length blots of Figure 4D, 4F, and 4H. (A) TRB3, (B) phosphorylated Akt, (C) total Akt, (D) phosphorylated mTOR, (E) total mTOR, (F) LC3, and (G) actin in Figure 4D. (H) LC3 and (I) actin in Figure 4F. (J) cleaved caspase3 and (K) actin in Figure 4H.

**Supplementary Figure 1**


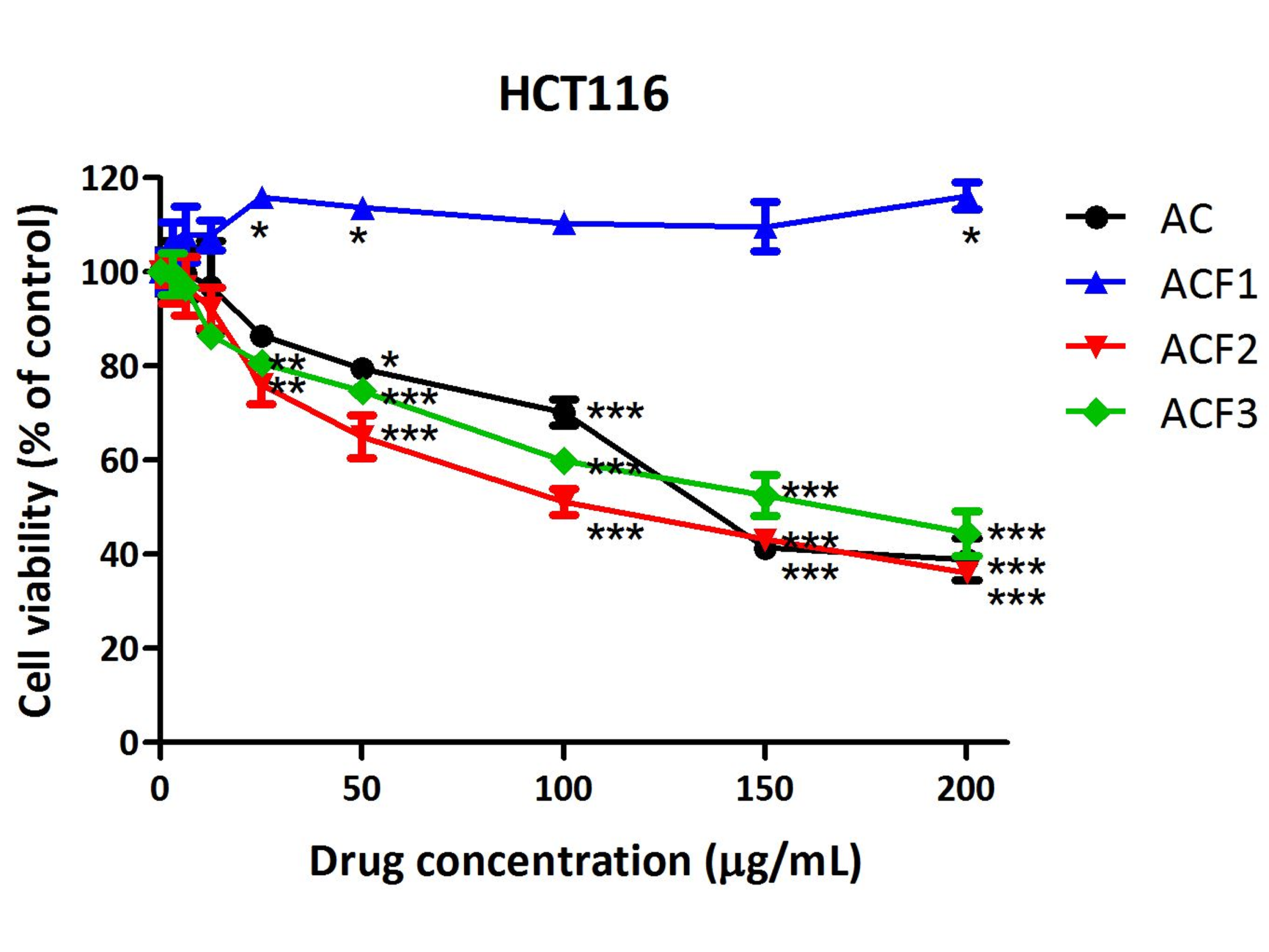


**Supplementary Figure 2**


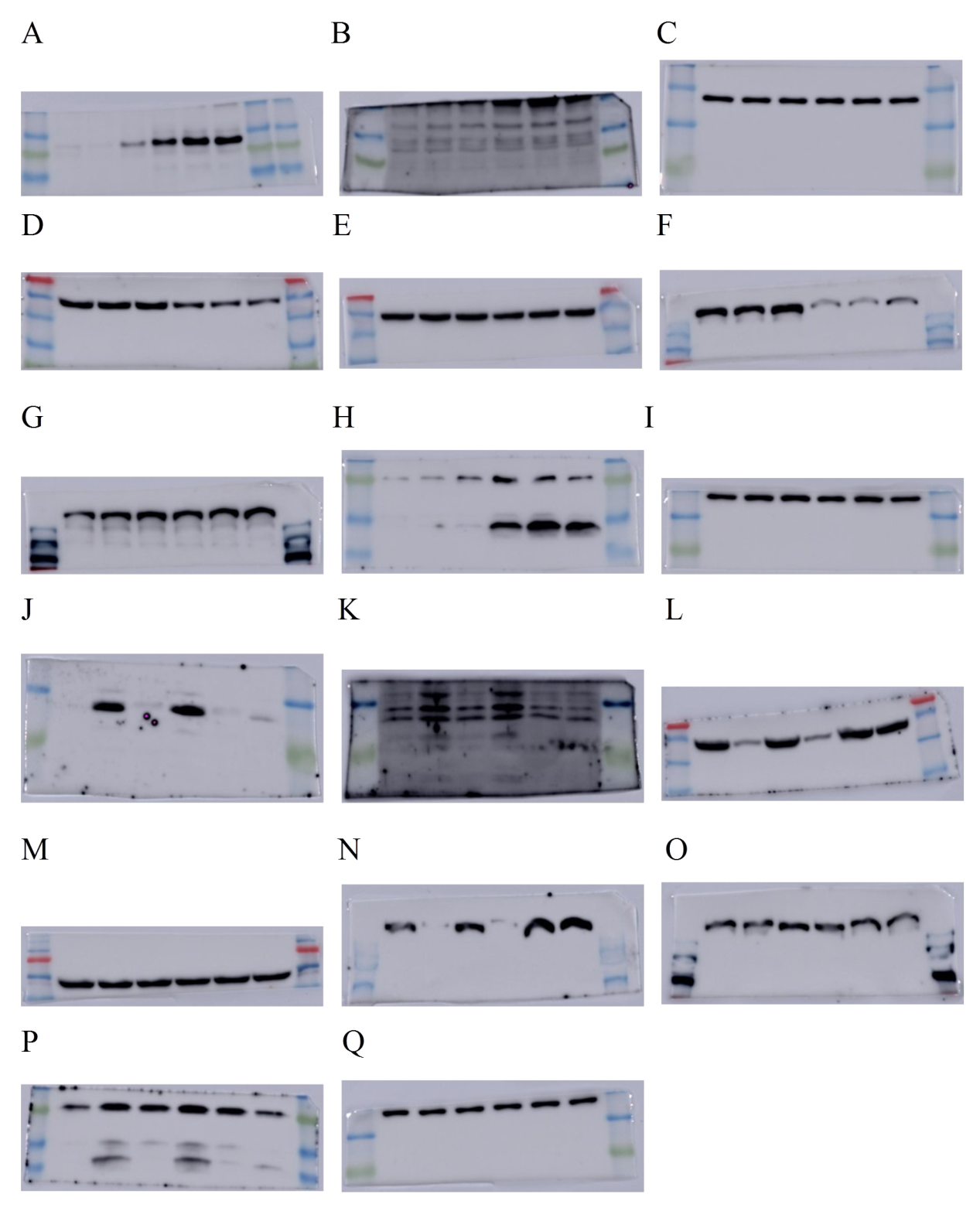


**Supplementary Figure 3**

**
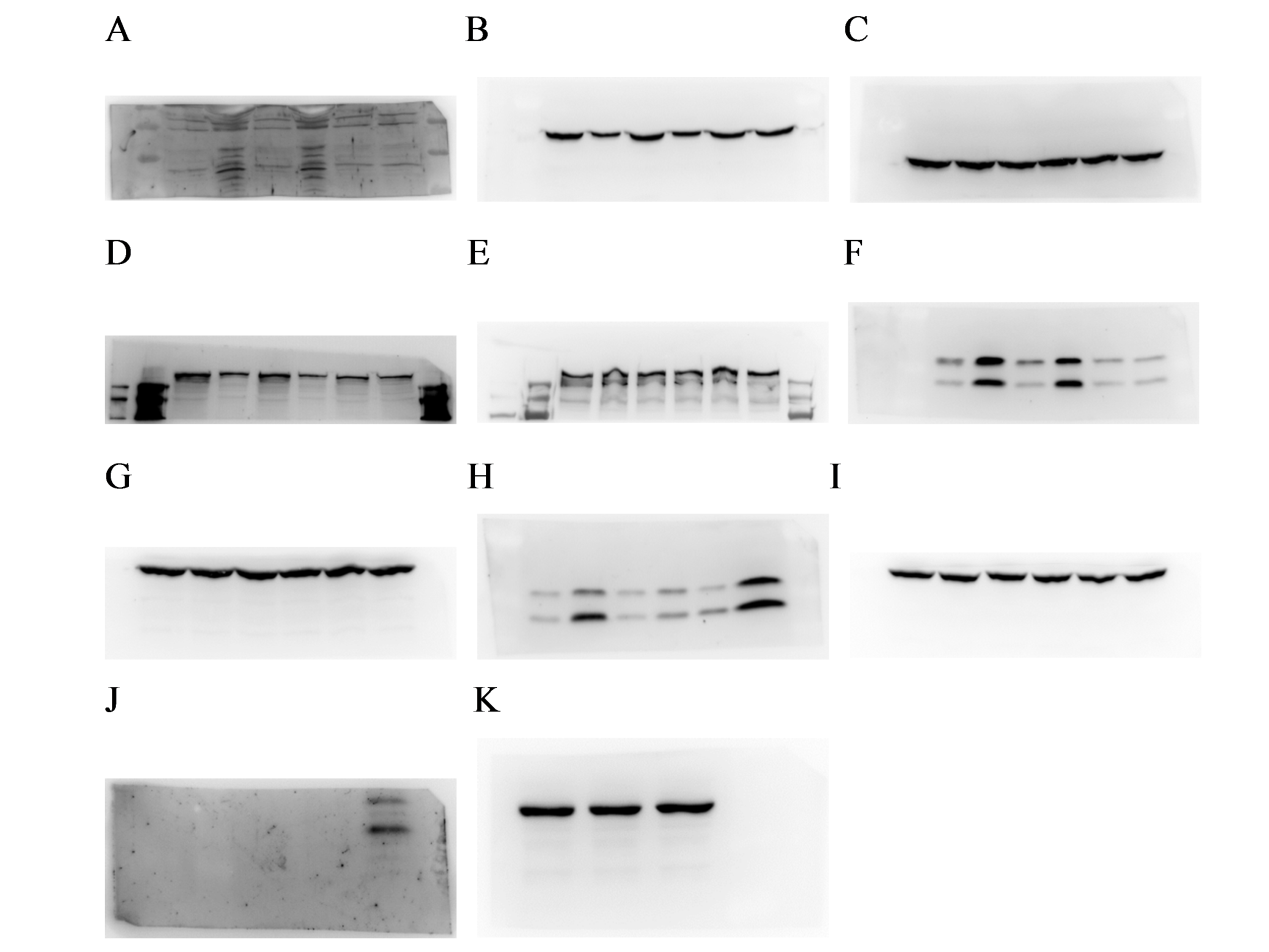
**
